# Social Isolation and Its Related Factors in Participants of a Senior Citizens’ College in Japan

**DOI:** 10.1093/geroni/igaa057.1027

**Published:** 2020-12-16

**Authors:** Hayato Uchida, Yoshinori Fujiwara, Shinro Matsuura

**Affiliations:** 1 University of Hyogo, Himeji, Hyogo, Japan; 2 Tokyo Metropolitan Institute of Gerontology, Tokyo, Japan; 3 Matsuura Clinic, Himeji, Hyogo, Japan

## Abstract

The aim of this study was to clarify the social isolation and its related factors of participants in senior citizen’s college in Japan. The participants were 104 persons (age 73.3+/-4.3) aged sixty-five years or over living in Hyogo Prefecture, Japan. They were the students of a senior citizens’ college established by Himeji City, Hyogo Prefecture in 1981. We conducted a cross- sectional study that included age, family structure, employment status, financial status, self-related health, presence of chronic disorders, instrumental activities of daily living, dietary variety score (1-10). The mental health well-being was assessed using the Japanese version of the World Health Organization Mental Health Well-being Index-five items (WHO-5). We defined social isolation as seeing friends or relatives less than two or three times a month. We carried out the survey in November in 2019. Selected variables were compared after dividing the participants into “socially isolated group” and “not socially isolated group”. A t-test, a chi-square test, and logistic regression analysis were conducted to examine the related factors of social isolation in this study. Of the 104 participants who were analyzed for social isolation, 21 (20.2%) were found to be socially isolated. From the results of multivariate logistic regression analysis, “lower mental health” (OR=4.013, 95%CI=1.745-10.435) and “lower self-related health” (OR=2.583, 95%CI=1.103-9.564) were independently associated with social isolation. These results suggest that, to prevent the social isolation of the participants in senior citizen’s college in Japan, it is necessary to develop and implement the approach which promote mental health and self-related health.

